# Medical studentś needs for an integration of climate change into the medical curriculum: Leonie Rybol

**DOI:** 10.1093/eurpub/ckac130.068

**Published:** 2022-10-25

**Authors:** L Rybol, D Amelung, J Nieder, H Hachad, A Depoux, R Sauerborn, A Herrmann

**Affiliations:** 1 Medical Faculty, Heidelberg Institute of Global Health, Heidelberg, Germany; 2 Medical Faculty, Sorbonne University, Paris, France; 3 Centre Virchow-Villermé of Public Health, University of Paris Cité, Paris, France

## Abstract

**Background:**

The impacts of climate change (CC) on health comprise increased human morbidity and mortality. Consequently, physicians need to be systematically trained to address CC in their professional life. Due to lacking research on educational needs of medical students, we developed a survey instrument to assess studentś attitudinal and knowledge-based needs for the integration of CC into medical curricula and their readiness to learn.

**Methods:**

Our survey was administered online to 788 students at the Medical Faculty of Heidelberg University between 06/2021 and 02/2022. Data analyses included descriptive statistics, reliability analyses as well as regression modeling with regard to readiness to learn.

**Results:**

214 students participated in the survey, 170 fully completed datasets were included in the analysis. A majority of students (72.35%) (strongly) agreed that doctors carry a responsibility to address CC in their work setting, while only 4.71% (strongly) agreed, that their current medical training had imparted them with enough skill to do so. Students showed both considerable knowledge and interest in the area of CC, its health impacts, vulnerabilities and clinical adaptation (70.09% correct answers). Knowledge gaps were identified in the areas of health co-benefits and sustainable healthcare (55.53% and 16.71% of correct answers). 79.42% of students want to learn about CC through the integration into existing mandatory courses.

**Conclusions:**

Results encourage the integration of CC topics with a focus on knowledge and professional role development into existing mandatory courses of the medical curriculum. Specifically, they also pinpoint health impacts and adaptation as greatest areas of interest for students and at health co-benefits and sustainable healthcare as areas with least prior knowledge.

**Key messages:**

- Heidelberg medical students want to learn about CC in their curricula.
- They have decent knowledge about CC and health but knowledge deficits in the topic areas of health co-benefits and green health sector.

